# A fatal case associated with respiratory syncytial virus infection in a young child

**DOI:** 10.1186/s12879-018-3123-8

**Published:** 2018-05-11

**Authors:** Lili Xu, Hengmiao Gao, Jiansheng Zeng, Jun Liu, Cong Lu, Xiaolei Guan, Suyun Qian, Zhengde Xie

**Affiliations:** 1 Beijing Key Laboratory of Pediatric Respiratory Infection Diseases, Key Laboratory of Major Diseases in Children, Ministry of Education, National Clinical Research Center for Respiratory Diseases, National Key Discipline of Pediatrics (Capital Medical University), Beijing Pediatric Research Institute, Beijing Children’s Hospital, Capital Medical University, National Center for Children’s Health, Beijing, China; 2 Department of Pediatric Critical Care Medicine, Beijing Children’s Hospital, Capital Medical University, National Center for Children’s Health, Beijing, China

**Keywords:** Respiratory syncytial virus, Central nervous system infection, Microbiome analysis

## Abstract

**Background:**

Respiratory syncytial virus (RSV) is the most common viral cause of pediatric bronchiolitis and pneumonia worldwide. Risk factors for high mortality and prolonged morbidity after RSV infection include premature birth, bronchopulmonary dysplasia, congenital heart disease, and Down syndrome. However, some previously healthy, full-term children who are infected with RSV also require hospitalization and even experience severe sequelae or death.

**Case presentation:**

In this report, we present the case of an RSV-associated death of a child who was born at full-term and developed normally up to the age of 2 years old. Cardiopulmonary arrest occurred within 3 days after the onset of symptoms, which included cough and high fever. Complete brain edema was prominent, and encephalopathy was developing. Viral antigen detection and microbiome analyses of oral swab and nasopharyngeal aspirate specimens verified an RSV infection, while bacterial culture of blood specimens yielded negative results. The RSV strain detected in this patient was subtyped as RSVB9, and no mutation was found in the six antigenic sites for targeted drugs or vaccines.

**Conclusions:**

The patient had a severe infection associated with RSV, which was very likely the cause of her central nervous system infection and acute neurological complications.

## Background

Respiratory syncytial virus (RSV) is the major cause of lower respiratory tract illness in children. For most children, an initial RSV infection normally occurred within the first 2 years of life. In infants less than 1 year of age and with lower respiratory infection, up to 80% are due to RSV [1]. In most cases, the virus is not fatal. The most severe infections and well-defined high-risk groups, including infants with a history of premature birth, and those with chronic lung disease, congenital heart disease, cystic fibrosis and immunodeficiency [2]. For those high-risk infants, palivizumab, a humanized monoclonal antibody which has produced favorable results to date, is strongly recommended to be administered prophylactically [3]. However, most children with RSV infection were previously healthy, and it is often difficult to predict deterioration of RSV infection [2, 4].

RSV-related encephalitis with acute encephalopathic symptoms such as seizure, severe sequelae and even death following RSV infection in children without underlying disease has sporadically been reported [5]. RSV-related encephalitis usually develops within 1 to 2 days after the onset of clinical symptoms, such as high fever, cough, and fatigue. However, the mechanism underlying the rapid progression of related encephalitis remains unclear. Central nervous system (CNS) infection, coinfection with bacteria, and dysfunction of the host immune system may be possible causes.

Next-generation sequencing (NGS) accompanied with metagenomic analysis can be used as a nonselective method for pathogen discovery and is increasingly applied as a diagnostic tool to investigate the causes underlying unexplained encephalitis in patients. Two key features of this methodology are: (1) it makes no assumptions about the type of pathogen and has the potential to detect nucleotides from all species, and (2) the causative pathogen may not necessarily be the most abundant signal in the NGS results and may sometimes be present as only a low-level signal compared to all other signals associated with commensal pathogens. It can, however, provide unbiased and sensitive identification.

In this report, we present the case of a 2-year-old girl who was not born prematurely and had no underlying disease whose sudden death may have been related to an RSV infection identified by conventional methods and metagenomic analyses.

## Case presentation

The patient in this fatal case was a 2-year-old girl who was born full-term and had developed normally. She had no medical history of asthma or pneumonia and no familial history of immunodeficiency. She had no brothers or sisters.

The patient was admitted to the hospital due to 3 days of fever and 15 min of respiratory and cardiac arrest. The symptoms started on 10 November (3 days before admission), with a fever (up to 39.8 °C) but no chills, rash or convulsions. Ibuprofen was given orally. The body temperature decreased for 4 to 5 h but then climbed to 40.3 °C. Shortness of breath accompanied the fever, and the body temperature did not decrease obviously after the oral administration of ibuprofen. Wheezing caused by the retention of phlegm in the throat and a single paroxysmal cough occurred. The patient occasionally coughed up a small amount of yellow phlegm and had a slightly runny nose. She had no asthma, breathing difficulty or hemoptysis; she had lethargy and a poor appetite but no vomiting or diarrhea. On 11 November (2 days before admission), the patient still had a high fever, and her body temperature fluctuated around approximately 40 °C. The patient’s body temperature did not obviously decrease after she was given oral ibuprofen and acetaminophen alternately. Acute infection was considered from then on. The patient received an intravenous infusion of 0.14 g Zithromax and 120 mg rographolide as well as aerosol inhalation of 2 mg budesonide, but her fever persisted, and her body temperature rose to a peak of 40 °C. On 12 November (1 day before admission), the patient still had a persistent fever and wheezing due to the retention of phlegm in her throat. She had shortness of breath, a light cough, and a drooping spirit, accompanied by a rash on the torso and limbs. Her appetite was slightly improved, and she had no vomiting or convulsions. On 13 November, the patient had sudden respiratory and cardiac arrest 3 h before admission. She was immediately and continuously treated with cardiopulmonary resuscitation by physicians and the intravenous injection of adrenaline (4 times). She was treated with trachea cannula and mechanical ventilation, and her heart beat recovered approximately 15 min later, but the patient remained in a deep comatose state with no spontaneous breathing. Then, the patient was transferred to our hospital and immediately underwent electrocardiogram (ECG) monitoring. Bloody fluid was visible in the indwelling gastrointestinal decompression tube, and the blood-gas analysis showed metabolic acidosis. The patient was treated with sodium bicarbonate to correct the acidosis. She was diagnosed with an acute CNS infection and brain hernia. After cardiopulmonary resuscitation, she was admitted to the pediatric intensive care unit (PICU). The head CT scan showed extensive brain swelling, decreased brain parenchymal density, narrowed cerebral ventricles and cisterns. These findings prompted a diagnosis of extensive brain edema and hernia. The chest posteroanterior radiograph showed fuzzy, coarse bilateral lung markings, visible small patchy shadows in the right inferior lung and a clear pulmonary hilus. These findings were diagnosed as pneumonia. Routine blood examination results suggested the presence of a bacterial infection; thus, the patient was treated with vancomycin and meropenem to control an infection. After that, immunoglobulin (1 g/kg) was administered for immune support. The patient was still in a deep coma state, and light reflexes of both pupils were absent. The patient’s spontaneous breathing was weak and irregular, and she had no response to painful stimulation. Compared with earlier, her rash was reduced, and the pulmonary lesions shown on the chest posteroanterior radiograph were slightly absorbed. Immunoglobulin (1 g/kg) was continuously administered to neutralize pathogens. On 15 November (3 days after admission), transcranial Doppler ultrasound assessment showed that the patient’s anterior and posterior cerebral circulation corresponded to the diagnostic criteria for brain death. On 17 November (5 days after admission), various organ functions failed, and the patient could not tolerate a spontaneous breathing test. Her guardian chose to quit treatment, and the patient died.

### Laboratory diagnosis

Viral antigen detection based on both an immunofluorescence assay and the Luminex xTAG respiratory viral panel assay was positive for RSV in the patient’s nasopharyngeal aspirates (which were collected on 14 Nov, the 5th day of disease onset and the 2nd day of admission) and negative for adenovirus, influenza A and B viruses, parainfluenza virus 1–4, human metapneumovirus, enteroviruses and rhinoviruses, human coronavirus HKU1, 229E, NL63 and OC43, and human bocavirus. Because the patient’s guardian refused to consent to lumbar puncture, cerebrospinal fluid (CSF) was not available for the detection of CNS infection.

The blood biochemistry results are summarized in Table 1. The amounts of red blood cells (RBCs), hemoglobin, and platelets continuously decreased after the onset of symptoms. Extremely high levels of C-reactive protein from the third day (36–104 mg/L) suggested viral or bacterial infection; however, bacterial cultures of blood specimens yielded negative results.

**Table 1:** Blood parameters and pathogen detection results of the patient

		D1 (Day of disease onset, 10th Nov)	D3 (12th Nov)	D4 (Day of admission, 13th Nov)	D5 (14th Nov)	D6 (Day of brain death, 15th Nov)	D7 (16th Nov)	D8 (Death, 17th Nov)	Normal range
Blood parameters	WBC [× 10^9^/L]	10.77	15.96	12.60	/	6.93	14.83	/	4.00–10.00
	RBC [× 10^12^/L]	4.87	4.16	3.89		3.29	2.8		3.50–5.50
	Hemoglobin [g/L]	130	111	104		89	73		110–160
	Platelets [× 10^9^/L]	236	116	128		87	41		100–400
	NEU %	65.8	73.7	54.9		50.8	48.6		18–46
	LYM %	24.3	14.6	35.6		33.5	37.4		37–78
	MONO %	9.0	11.6	8.9		13.7	13.2		3.0–10.0
	EOS %	0.9	0	0.5		1.6	0.5		0.5–5.0
	CRP [mg/L]	1.4	36	73		104	82		< 8
Pathogen detection	Bacterial culture	/	/	(−)	/	(−)	/	(−)	(−)
	Viral antigen and molecular detection	/	/	/	RSV(+)	/	/	/	(−)

*Abbreviations*: *WBC* white blood cell, *RBC* red blood cell, *NEU* neutrocyte, *LYM* lymphocyte, *MONO* monocyte, *EOS* eosinophil, *CRP* C-reactive protein, (−) negative, (+) positive

The percentage of T lymphocytes was 46.6%, of which helper T cells and suppressor T cells accounted for 29.6 and 13.2%, respectively. The ratio of CD4/CD8 was 2.2. The proportions of B lymphocytes and NK cells were 45.6 and 3%, respectively. All these immunological indexes indicated dysfunction of the patient’s immune system.

### Metagenomic and viral molecular analysis

Oral swab, nasopharyngeal aspirate, and serum specimens collected on 14 Nov (the 5th day of disease onset and the 2nd day of admission) were subjected to multiplex metagenomic analyses using an NGS platform. The nucleic acid library was constructed as previously described [6]. The amplified nucleic acid libraries were then analyzed using an Illumina HiSeq 4000 sequencer for a single read of 126 bp. The raw sequence reads were filtered using previously described criteria [7] to obtain valid sequences.

**Fig. 1:**
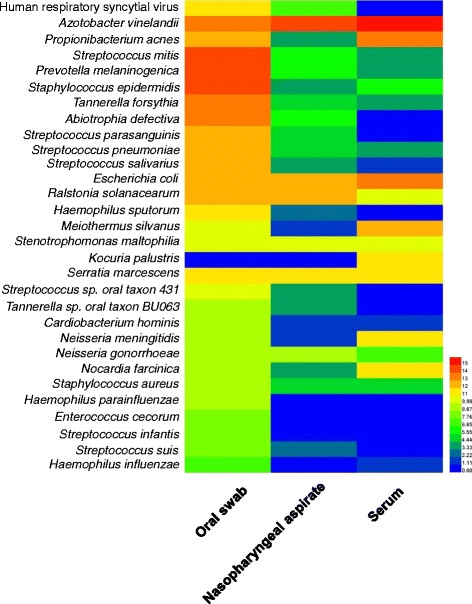
Heatmap based on the read numbers of pathogens derived from the oral swab, nasopharyngeal aspirate, and serum specimens. The clinical specimens are listed in the bottom row, and the pathogen names are presented in the left column. The boxes colored from blue to red represent the metagenomic sequencing reads observed (the reads varied between 2^0^ and 2^15^)

When bacteriophages, plant-origin sequences resulting from food debris in the oral cavity, and contamination from the reagents used in the sample processing step (murine leukemia virus (MLV), for example) were excluded, only human RSV (based on the NCBI taxonomy database) was identified, with at least one specific sequence from the oral swab (2137 reads) and the nasopharyngeal aspirate (146 reads). No virus-related sequences were detected in the serum specimen. Meanwhile, large numbers of sequence reads related to bacteria, including *Streptococcus mitis, Streptococcus parasanguinis, Streptococcus pneumoniae, Streptococcus salivarius, Streptococcus infantis*, *Streptococcus suis, Neisseria meningitidis, Neisseria gonorrhoeae, Haemophilus sputorum, Haemophilus parainfluenzae, Enterococcus cecorum,* and other conditioned pathogenic bacteria were also detected in the oral swab, nasopharyngeal aspirate, or/and serum specimens (Fig. 1). However, although the metagenomic analysis showed sequence reads assigned to Kingdom Bacteria, the bacterial culture of the blood specimens yielded negative results.

**Fig. 2:**
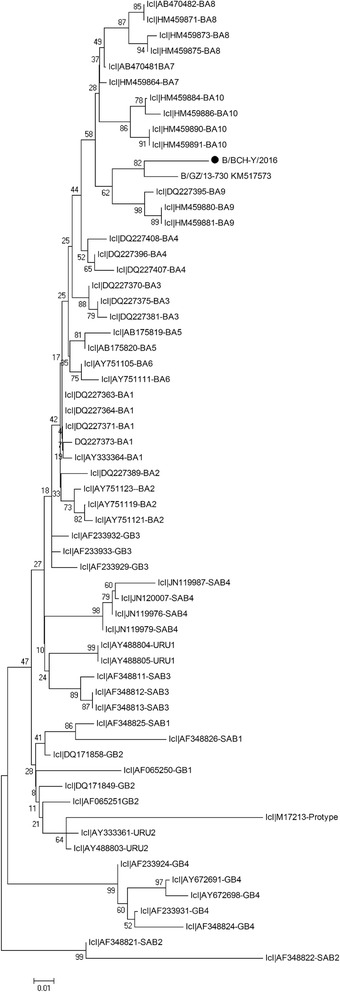
Phylogenetic analysis based on the complete G gene sequence of the RSV detected in this patient and other representative sequences from nearly all RSVB subgroups (BA1–10, GB1–4, SAB1–4, URU1–2) from GenBank. The nucleotide phylogenetic tree was constructed using the neighbor-joining method with nucleotide p distances and 1000 bootstrap replicates in the Molecular Evolutionary Genetics Analysis program (MEGA, version 4.0, USA)

Based on the random distribution of reads of the RSV virus genome, the complete length of the genome was obtained using NGS methods and gap amplification. This RSV strain was subtyped as RSVB; it was found to cluster in the BA genotype and had the signature 60-bp duplication in the G gene. The newly identified virus was named RSVB/BCH-Y/2016, and the full genome sequence was deposited in GenBank under accession number KY924878. The phylogenetic analysis was conducted with representative sequences from nearly all RSVB subgroups (BA1–10, GB1–4, SAB1–4, URU1–2) from GenBank; RSVB/BCH-Y/2016 belonged to BA9 subgroup (Fig. 2). The nucleotide homology comparisons revealed that the G gene of this strain was most closely related (share 98.82% homology) to strain RSVB/GZ/13–730, which was isolated from a child in Guangzhou, China, in 2013. For the six most important antigenic sites (Ø, I, II, IV, V, VI) in the fusion protein for drug or vaccine (such as palivizumab) targeting [8, 9], no mutation was found in RSVB/BCH-Y/2016.

## Discussion and conclusions

RSV infection generally causes symptoms such as fever, cough and wheezing. While respiratory failure may occur in severe cases, the fatality rate is not high in clinical cases. The patient in this case study was infected with RSV, and the disease progressed rapidly, with extensive cerebral edema and hernia.

There are prior reports of neurological complications of RSV infection, which mainly include central apnea, seizures, and encephalopathy [10]. Apnea is a common occurrence in RSV-infected patients younger than 2 months of age and is a frequent indication for intubation [11, 12]. The mechanism of RSV-related apnea is unknown, but a significantly reinforced laryngeal chemoreflex or an immature respiratory center in infancy has been proposed [11, 13]. Evidence of an encephalitic pathology caused by RSV infection is sparse. The presence of RSV in CSF, detected using polymerase chain reaction, was reported in a single case of a 4-month-old boy with febrile seizure [14]. The pathogenesis of RSV-related encephalitis is not yet fully understood. However, it has been hypothesized that RSV may enter the CNS through the hematogenous/blood-brain barrier route or through invasion, causing the release of several humoral neurotoxic cytokine mediators [15].

The diseases that we considered in the course of treatment included the following: (1) Severe hand, foot and mouth disease (usually caused by enterovirus, type 71): this disease has a sudden onset in most cases, and symptoms include fever, rashes and herpes appearing on the oral mucosa, hands, and feet. This disease progresses quickly in a small number of cases, especially in children younger than 3 years old. At 1–5 days after onset, complications such as meningitis, encephalitis, encephalomyelitis, pulmonary edema and circulatory disorders occur. A very small number of infections worsen and lead to death. However, symptoms such as herpes and pulmonary edema were not observed in this patient, and both viral pathogen analyses showed negative results for enteroviruses, so this speculation was not supported. (2) High pathogenic influenza virus infection: the typical symptoms include fever, cough, sore throat, headache, body pain and fatigue. Some patients progress quickly to severe pneumonia, acute respiratory distress syndrome (ARDS), shock and acute necrotic brain injury. However, this child underwent repeated influenza A virus antigen detection, and all results were negative; thus, this speculation was not supported. (3) Fulminant myocarditis: the initial symptoms primarily include fever, cough, fatigue and vomiting. The disease develops rapidly and can lead to sudden congestive heart failure, cardiogenic shock, and Adam-Strokes syndrome. ECG sometimes demonstrates ST-T changes, myocardial infarction, and arrhythmia. The cardiac ultrasound may show left ventricular enlargement. However, no abnormalities were observed in the ECG or heart ultrasound analyses of our patient; thus, this speculation was not supported.

Because the patient’s guardian refused to consent to lumbar puncture, a CSF specimen was not available for further validation. We could not be sure whether the neuroinvasion of RSV did occur in this case because RSV has only rarely been identified in CNS specimens [12]. This finding is somewhat similar to that of influenza encephalitis. Britton et al. have described cases of influenza-associated neurological disease without testing for influenza viruses in CSF specimens [16]. Whether the influenza virus invades the CNS or not is still controversial. Fujimoto et al. reported that influenza virus RNA was detected in the CSF of 71.4% (5/7) of patients who developed influenza-associated acute encephalopathy/encephalitis [17]. However, in other reports, only a small number of patients were positive for viral RNA in the CSF and brain, and there was a lack of inflammation in the brain tissue of fatal cases [18–21]. In the reported cases, no influenza RNA was detectable in the CSF. However, this finding does not exclude influenza as a cause of the encephalitis because the viral RNA may have been cleared by the time the CSF was taken. The fact that the RNA is no longer detectable does not exclude an earlier CNS insult by the virus causing the current symptoms. For the case reported here, although direct evidence of RSV infection in the CNS was not available, the clinical symptoms together with the laboratory findings and metagenomic analysis results suggested that the patient may have had severe sepsis that potentially resulting from an RSV infection with a high probability of CNS infection and acute neurological complications.

Combining NGS with metagenomic analysis provides an important clinical tool to identify unexpected or novel pathogens in patients. The advantage of this methodology is maximized when the causative pathogen presents a low-level signal compared to all other signals representing environmental and commensal pathogens. Improving the optimization and implementation of protocols suitable for clinical samples will no doubt improve microbial diagnosis in clinical practice.

Our findings, in conjunction with previously reported cases emphasize the need for more awareness of the neurological complications of RSV infection. Its clinical manifestations may include seizures, encephalopathy, and focal neurological findings. Early recognition of neurological complications of RSV infection is important for initiating effective treatment to reduce mortality and long-term morbidity.

## Abbreviations

ARDS: Acute respiratory distress syndrome
CNS: Central nervous system
CRP: C-reactive protein
CSF: Cerebrospinal fluid
ECG: Electrocardiogram
EOS: Eosinophil
LYM: Lymphocyte
MLV: Murine leukemia virus
MONO: Monocyte
NEU: Neutrocyte
NGS: Next-generation sequencing
PICU: Pediatric intensive care unit
RBC: Red blood cell
RSV: Respiratory syncytial virus
WBC: White blood cell

## References

[1] Piedimonte G (2015). RSV infections: state of the art. Cleve Clin J Med.

[2] Hall CB, Weinberg GA, Iwane MK, Blumkin AK, Edwards KM, Staat MA, Auinger P, Griffin MR, Poehling KA, Erdman D (2009). The burden of respiratory syncytial virus infection in young children. N Engl J Med.

[3] Brady MT, Byington CL, Davies HD, Edwards KM, Jackson MA, Maldonado YA, Murray DL, Orenstein WA, Rathore MH, Sawyer MH (2014). Updated guidance for Palivizumab prophylaxis among infants and young children at increased risk of hospitalization for respiratory syncytial virus infection. Pediatrics.

[4] Brooks AM, McBride JT, McConnochie KM, Aviram M, Long C, Hall CB (1999). Predicting deterioration in previously healthy infants hospitalized with respiratory syncytial virus infection. Pediatrics.

[5] Morichi S, Morishita N, Ishida Y, Oana S, Yamanaka G, Kashiwagi Y, Kawashima H (2017). Examination of neurological prognostic markers in patients with respiratory syncytial virus-associated encephalopathy. Int J Neurosci.

[6] Wang Y, Zhu N, Li Y, Lu R, Wang H, Liu G, Zou X, Xie Z, Tan W (2016). Metagenomic analysis of viral genetic diversity in respiratory samples from children with severe acute respiratory infection in China. Clin Microbiol Infec.

[7] Wu ZQ, Yang L, Ren XW, He GM, Zhang JP, Yang J, Qian ZH, Dong J, Sun LL, Zhu YF (2016). Deciphering the bat virome catalog to better understand the ecological diversity of bat viruses and the bat origin of emerging infectious diseases. Isme J.

[8] McLellan JS, Chen M, Leung S, Graepel KW, Du X, Yang Y, Zhou T, Baxa U, Yasuda E, Beaumont T (2013). Structure of RSV fusion glycoprotein trimer bound to a prefusion-specific neutralizing antibody. Science.

[9] McLellan JS (2015). Neutralizing epitopes on the respiratory syncytial virus fusion glycoprotein. Curr Opin Virol.

[10] Eisenhut M (2006). Extrapulmonary manifestations of severe respiratory syncytial virus infection--a systematic review. Crit Care.

[11] Kneyber MC, Brandenburg AH, de Groot R, Joosten KF, Rothbarth PH, Ott A, Moll HA (1998). Risk factors for respiratory syncytial virus associated apnoea. Eur J Pediatr.

[12] Millichap JJ, Wainwright MS (2009). Neurological complications of respiratory syncytial virus infection: case series and review of literature. J Child Neurol.

[13] Lindgren C, Grogaard J (1996). Reflex apnoea response and inflammatory mediators in infants with respiratory tract infection. Acta Paediatr.

[14] Zlateva KT, Van Ranst M (2004). Detection of subgroup B respiratory syncytial virus in the cerebrospinal fluid of a patient with respiratory syncytial virus pneumonia. Pediatr Infect Dis J.

[15] Park A, Suh SI, Son GR, Lee YH, Seo HS, Eun BL, Lee NJ, Seol HY (2014). Respiratory syncytial virus-related encephalitis: magnetic resonance imaging findings with diffusion-weighted study. Neuroradiology.

[16] Britton PN, Blyth CC, Macartney K, Dale RC, Li-Kim-Moy J, Khandaker G, Crawford NW, Marshall H, Clark JE, Elliott EJ (2017). The Spectrum and burden of influenza-associated neurological disease in children: combined encephalitis and influenza sentinel site surveillance from Australia, 2013-2015. Clin Infect Dis.

[17] Fujimoto S, Kobayashi M, Uemura O, Iwasa M, Ando T, Katoh T, Nakamura G, Maki N, Togari H, Wada Y (1998). PCR on cerebrospinal fluid to show influenza-associated acute encephalopathy or encephalitis. Lancet.

[18] Togashi T, Matsuzono Y, Narita M, Morishima T (2004). Influenza-associated acute encephalopathy in Japanese children in 1994-2002. Virus Res.

[19] Evans AS, Agadi S, Siegel JD, Chung WM, Carlo JT, Uyeki TM, Sejvar J, Lindstrom S, Erdman D, Oberste S (2009). Neurologic complications associated with novel influenza a (H1N1) virus infection in children-Dallas, Texas, May 2009 (reprinted from MMWR, vol 58, pg 773-778, 2009). Jama-J Am Med Assoc.

[20] Morishima T, Togashi T, Yokota S, Okuno Y, Miyazaki C, Tashiro M, Okabe N, Influenza CSG (2002). Encephalitis and encephalopathy associated with an influenza epidemic in Japan. Clin Infect Dis.

[21] Ito Y, Ichiyama T, Kimura H, Shibata M, Ishiwada N, Kuroki H, Furukawa S, Morishima T (1999). Detection of influenza virus RNA by reverse transcription-PCR and proinflammatory cytokines in influenza-virus-associated encephalopathy. J Med Virol.

